# Swelling Behavior of Anionic Hydrogels: Experiments and Modeling

**DOI:** 10.3390/gels10120813

**Published:** 2024-12-10

**Authors:** Raffaella De Piano, Diego Caccavo, Anna Angela Barba, Gaetano Lamberti

**Affiliations:** 1 Department of Industrial Engineering, University of Salerno, 84084 Fisciano, Italy; rdepiano@unisa.it (R.D.P.); glamberti@unisa.it (G.L.); 2 Department of Pharmacy, University of Salerno, 84084 Fisciano, Italy; aabarba@unisa.it

**Keywords:** polyelectrolytes, anionic hydrogels, pH, ionic strength, modeling

## Abstract

Polyelectrolyte hydrogels are smart materials whose swelling behavior is governed by ionizable groups on their polymeric chains, making them sensitive to pH and ionic strength. This study combined experiments and modeling to characterize anionic hydrogels. Mechanical tests and gravimetric analyses were performed to track hydrogel mass over time and at a steady state under varying pH and salt concentrations. The swelling ratio exhibited a bell-shaped curve with pH, reaching 120 in pure water, and decreased with increasing salt concentrations. Transient regimes showed slower swelling (~40 h) under pH stimulation compared to faster deswelling (~20 h) induced by salt. A fully coupled model integrating mass transport and solid mechanics was developed, with solvent diffusivity as the sole adjustable parameter in transient simulations. In conclusion, this study combined experiments and modeling to uncover complex mechanisms in PE behavior under two external stimuli, providing insights essential for designing advanced hydrogels.

## 1. Introduction

Hydrogels are versatile materials that can absorb and desorb water, causing them to swell and shrink [1,2]. Smart hydrogels, a subset of these materials, are particularly interesting due to their sensitivity to external stimuli such as mechanical stress, electric or magnetic fields, ionic strength and pH [3,4,5]. These smart, sensitive materials are fundamental to a variety of applications, particularly in the biomedical field [6,7,8,9,10,11,12,13,14]. As deeply examined in the work of Cascone and Lamberti [15], they are used in the formulation of drug delivery devices, transdermal drug delivery systems, tissue engineering and wound dressing applications.

Polyelectrolyte hydrogels are an example of smart hydrogels. They contain, in fact, ionizable functional groups (acid or basic) that can dissociate or associate, depending on the solution’s pH and the group’s specific pKa. This property allows the polyelectrolyte to expand or contract their polymeric chains in response to changes in pH and ionic strength. Anionic polyelectrolyte hydrogels, for instance, demonstrate complex pH-dependent behavior at the steady-state, as discussed in our previous work [16]. At low pH, the groups remain protonated, limiting swelling. As the pH increases towards the pKa, dissociation occurs, leading to a chain expansion and subsequent water absorption. However, at higher pH values, counterions (e.g., Na^+^) can interact with the dissociated groups, leading to neutralization and subsequent hydrogel shrinkage.

While the role of pH is relatively straightforward, the role of ionic strength is more complex and can vary significantly between different systems [17,18,19,20].

In this scenario, mathematical modeling, alongside experimental studies, is a crucial tool for comprehensively understanding the behavior of polyelectrolyte hydrogels. Over the years, various mathematical approaches, ranging from empirical to mechanistic models have been explored to describe hydrogel behavior. Readers interested in a comprehensive review of mathematical models for hydrogels can refer to the work of Caccavo [21].

Recently, our research groups proposed a “fully coupled” [22] model that considers the mutual influence of mass transport and mechanics. This model has been applied to both neutral and polyelectrolyte hydrogels [16,23,24].

Building upon our previous work and following the work of Marcombe [25], it has been established that polyelectrolyte hydrogels, unlike neutral hydrogels, are significantly influenced by ions and functional groups, in addition to mechanical and solvent transport. The swelling behavior of these materials is governed by a complex interplay of elastic forces, entropic mixing and entropic/enthalpic contribution arising from the presence of ions and functional groups in solution. Our previous modeling studies on polyelectrolyte hydrogels have primarily focused on steady-state behavior at pH values up to pH 7 and 14.

To delve deeper into this knowledge, this study aims to investigate the impact of pH and ionic strength on both the steady state and unsteady behavior of these materials.

A combination of experimental techniques (mechanical tests and gravimetric analysis) and modeling studies (based on an extended and modified version of (De Piano et al. [16,24]), will be adopted to explore the behavior of an anionic hydrogel.

For the first time in the literature, to the best of the authors’ knowledge, a fully coupled model will be used to describe the behavior of sensitive hydrogels in a steady state and in a transient regime simulation with two different external factors.

## 2. Results and Discussion

In the following section, the hydrogel’s behavior is discussed, from both experimental and modeling perspectives. The mechanical behavior is first investigated, allowing for the determination of the elastic modulus of the beads. Subsequently, the equilibrium (steady state) behavior is examined by varying the pH and the ionic strength, and the model parameters related to the polymer are obtained. Finally, these parameters are used to describe hydrogels’ transient behavior, in which only the parameter related to the solvent diffusivity is optimized.

### 2.1. Mechanical Compression Tests

**Figure 1:**
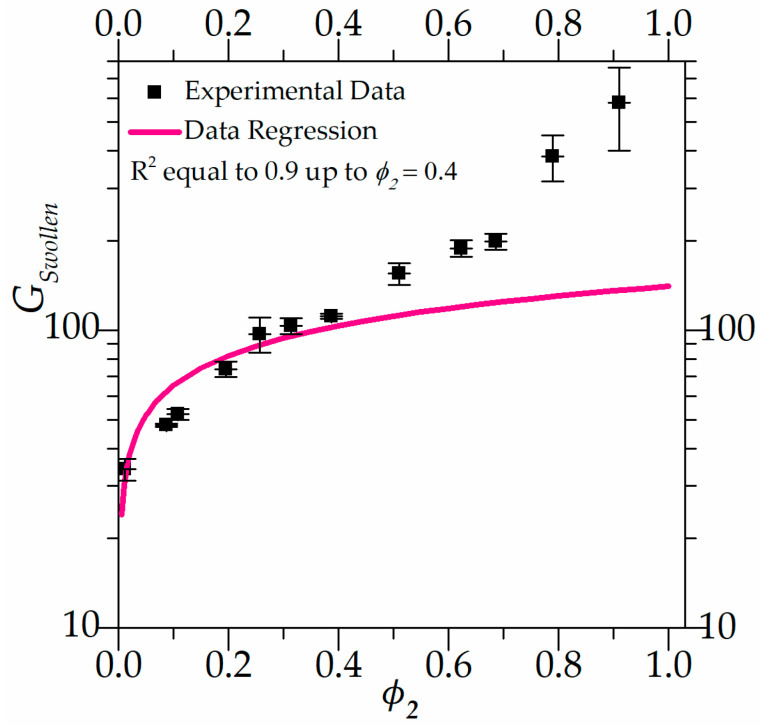
Mechanical compression results.

Figure 1 reports the result of the compression test; the elastic modulus of the swollen hydrogels for each polymer volume fraction is reported. *G_swollen_* is calculated from the Hertzian theory equations, as described in the previous section. *ϕ_2_* is related to the polymer volume fraction.

Experimental data are fitted through the equation that links the shear modulus of the swollen beads to the shear modulus of the dry hydrogel (*G_Dry_*) according to the equation $G_{Swollen}=G_{Dry}{\phi_{2}}^{1/3}$. This relation derives from the theory of rubber elasticity and is used for elastic materials. The behavior of elastic material can be observed, for this system, until the polymer fraction is equal to 0.4. For values higher than 0.4, the deviation from ideality is evident: the network elasticity cannot be described by the Gaussian elasticity theory. Values of polymer volume fraction higher than 0.4 indicated a situation in which the gel is not in the rubber state (glassy state) and the equation can no longer be used [17].

This result can be interpreted using fractal theory to determine the fractal dimension [26], which relates the shear modulus to the hydration level ((*ϕ_2_*)) of the system. Using the direct method: ${ln(G}_{Swollen})=\ln\left(G_{Dry}\right)+n\ln\left(\phi_{2}\right)$, where *n* is the fractal dimension. Regression analysis performed in the range *ϕ_2_* ∈ [0,0.4] yields *n* = 0.348 (R^2^ = 0.87), which is very close to the theoretical value of 1/3, as expected. In the other range, *ϕ_2_* ∈ [0.4,1], *n* = 2.377 (R^2^ = 0.89), indicating a significantly different microstructure compared to the rubber-like state analyzed in the earlier case (*ϕ_2_* ∈ [0,0.4]). From the intercept of the linear regression performed in the range *ϕ_2_* ∈ [0,0.4], the shear modulus of the dry polymer can be obtained: $G_{Dry}\left(constant\right)=140\;kPa$.

The value of $G_{Dry}\left(constant\right)=140\;kPa$ will be used in the next part of the work.

### 2.2. Steady-State Behavior

**Figure 2:**
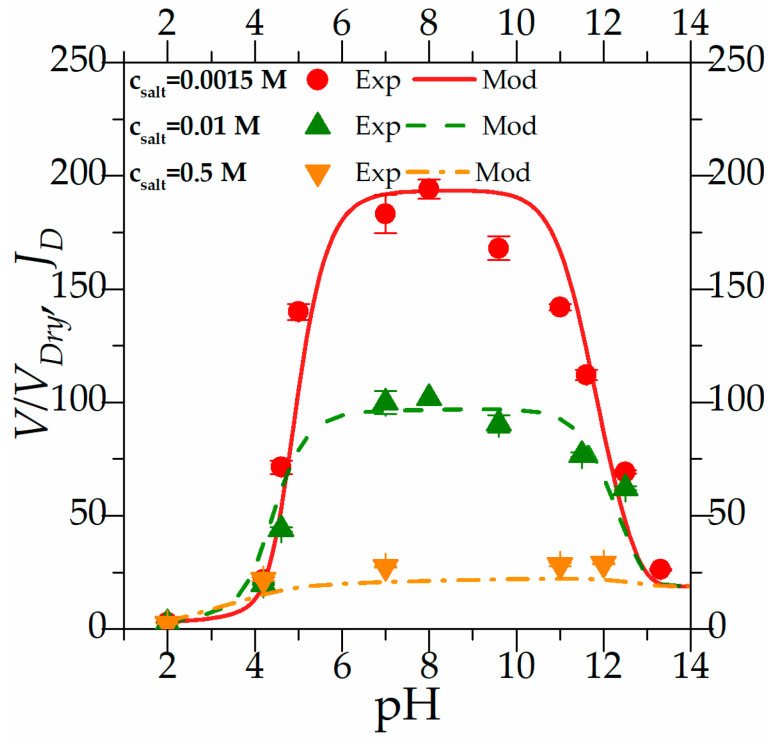
Steady-state behavior of anionic hydrogels’ beads. The experimental data are the equilibrium point calculated as *V*/*V_Dry_* for the full range of pH and three different concentrations of salt.

**Figure 3:**
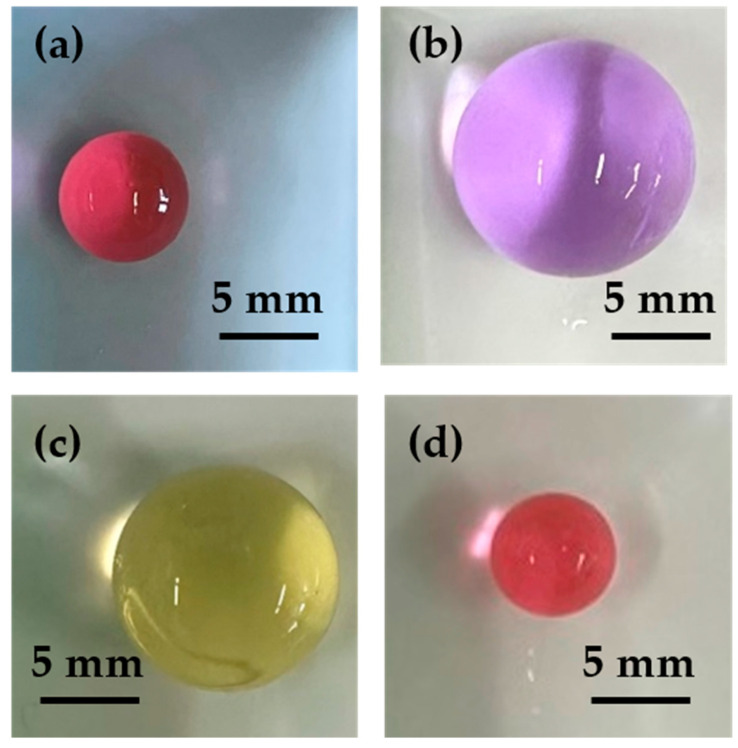
Equilibrium points for four different final pH conditions: (**a**) pH = 4.2; (**b**) pH = 7; (**c**) pH = 11; (**d**) pH = 13.

Figure 2 depicts the general behavior of an anionic hydrogel across the full range of pH from 2.0 to 14.0. Hydrogel beads were placed in solution with varying pH and ionic strength, and the experimental points represent the values of *V*/*V_Dry_* recorded under different equilibrium conditions. The complete behavior was recorded for three different salt concentrations. Figure 3 shows images of the hydrogels at a steady state for four different external pH values: (a) pH = 4.2, (b) pH = 7.0, (c) pH = 11; (d) pH = 13.

Focusing on the experimental results plotted in Figure 2, the general behavior of anionic polyelectrolyte hydrogels can be observed. Looking at the data for a salt concentration of 0.0015 M, three important focal points could be recognized: the “fully associated limit” at low pH, the “fully dissociated limit” around pH 7.0, and a “reassociated limit” at a high pH.

At a low pH, hydrogel beads are in equilibrium with an external solution far below the acidic dissociation “constant” and with a high concentration of protons “H^+^”. Consequently, the functional groups on the polymeric chains exist in their associated form as the acid group “AH”, representing the “fully associated limit”, which is maintained until the pH approaches 4.0.

As the external pH reaches the value of the acidic dissociation constant, the functional group “AH” begins to dissociate, generating protons “H^+^” and fixed charge “A^−^”, following the equilibrium reaction reported in Equation (3a). The polymeric chains start to extend, and the solvent diffuses into the matrix. At the same time, the external chemical potential of the solvent increases, driving water absorption and causing the hydrogel to swell. This behavior continues until pH 7.0, where all the functional groups are dissociated, marking the “fully dissociated limit”. This state persists from pH 7 until pH 10.

Beyond a pH of 7, sodium ions (from sodium hydroxide) are in solution and react with the dissociated groups “A^−^”, forming reassociated groups “ANa”, following the association reaction reported in Equation (3b). The polymeric chains start to contract, and, together with the decrease in the chemical potential of the external solvent, the hydrogel starts to desorb water and shrink, reaching the “reassociated limit” at pH levels approaching 14.

When moving from a low salt concentration to a higher one, the same behavior with the same three phases is observed, although it becomes less pronounced as the ionic strength increases. The external solvent chemical potential abruptly decreases, increasing the ionic strength, and the hydrogel tends to desorb water. The “fully dissociated limit”, which represents the maximum value of the swelling ratio, reaches a lower value than in lower salt concentrations. At a salt concentration of 0.5 M, the impact of the external solvent chemical potential (calculated as −42 J/mol) is predominant over the dissociation phenomenon, and the impact on the swelling is minimal.

Adding counter ions to a solution can impact polymer swelling in multiple ways. Beyond altering the external solvent chemical potential, counter ions can shield the fixed charge on the polymer chain, reducing electrostatic repulsion and leading to chain contraction and decreased swelling. It is important to note that the influence of ionic strength on swelling behavior is primarily observed in the “fully dissociated limit”. In contrast, at very low or very high pH, where ion association Equation (3a,b) predominate, ionic strength has a minimal effect on swelling. In this case, the equilibrium behavior is dominated by the concentrations of protons “H^+^” and hydroxide anions “OH^−^”, which predominate over-the-counter ions. This behavior is consistent with the literature data, as shown by Ostroha et al., 2004 [27].

In Figure 2, together with the experimental results, the modeling results are reported. The model, whose equations are described in the previous section, uses six parameters to fully characterize the problem: the acidic dissociation constant *K_a_*, the association constant *K_Na_*, the elastic modulus of the dry hydrogel *G_Dry_*, the number of the functional groups *f* and the Flory Huggins parameter *χ*_12_. The association constant is a parameter taken from reference [15], the elastic modulus is taken from the independent mechanical test discussed in the first part of the results, and the others are optimization parameters.

The value of the dissociation constant *K_a_* is related to the pKa of the acid group through *K_a_* = 10^−*pKa*^. In polyelectrolytes, the pKa is not simply equal to that of its constituent monomer but can be influenced by various factors like the polymer’s environment, the ionic strength of the solution, and even the polymer’s own structure [28,29]. For this reason, a medium value of acidic dissociation constant pKa was chosen as equal to 3.8.

The polymer–solvent interaction strongly depends on the pH condition. This dependency is accounted for by including the internal pH in the expression of the Flory Huggins parameter *χ*_12_. As explained by De Piano et al., 2023 [24], recalling Teraoka, 2002 [30] and Flory, 1953 [1], the Flory–Huggins parameter derives from the “Flory–Huggins mean-field theory” and is an indicator of the level of polymer–solvent interaction. For a dilute solution, the value close to ideality is 0.5. Values lower than 0.5 and higher than 0.5 represent, respectively, a favored and unfavored polymer–solvent interaction. In this case, the Flory–Huggins parameter was related to the internal pH through the following formula, where *α* and *β* become the optimization parameters:

(1) $$\chi_{12}=\frac{\alpha \cdot {pH}_{int}}{{pH}_{int}-\beta }$$

Parameters used are summarized in Table 1.

Parameters were optimized for the set of experimental data related to the first concentration of salt by automatically minimizing the Sum of the Squared Errors (SSEs) between the experimental and modeling results, as described in the modeling section. These parameters were then applied to the other experimental sets without any further adjustment. As can be observed from Figure 2, the model can depict the general behavior of the gel by describing the limits described before and all the transition zones. When the model is applied without modifications to the other experimental data, it continues to accurately represent the general behavior under different conditions. Thus, the model has proven to be not only descriptive but also predictive of the general behavior of anionic hydrogels under varying external salt concentrations.

### 2.3. Transient Regime

**Figure 4:**
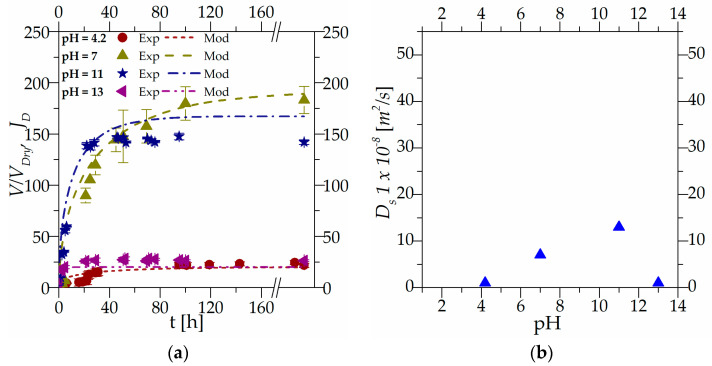
(**a**) Transient regime behavior obtained by changing the final pH conditions for a salt concentration equal to 0.0015 M, (**b**) solvent diffusivity versus final pH: $D_{s}$(pH) at constant salt concentration (0.0015 M).

Figure 4a shows the transient regime behavior of the hydrogel’s beads equilibrated at pH 2.0 (initial condition) and a salt concentration equal to 0.0015 M and then transferred in solutions at the same salt concentration but with pH equal to 4.2, 7.0, 11.0, and 13.0, respectively, representing the boundary conditions. Figure 4b is a representation of solvent diffusivity versus the final pH: $D_{s}$(pH) at a constant external salt concentration (0.0015 M).

**Figure 5:**
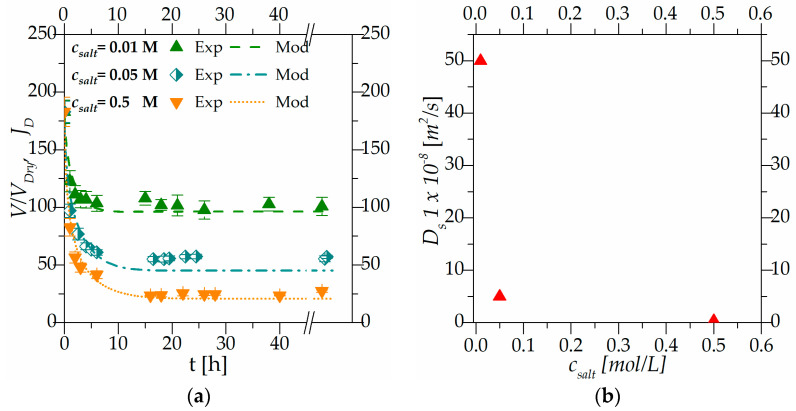
(**a**) Transient regime behavior at pH equal to 7.0 obtained by varying the external condition of salt, (**b**) solvent diffusivity versus final salt concentration: $D_{s}$(*c_salt_*) at a constant external pH (7).

Figure 5a, similar to Figure 4a, shows the transient regime behavior for the hydrogel’s beads equilibrated at pH 7.0 (initial condition) and transferred in solutions at the same pH but with salt concentrations equal to 0.01 M, 0.05 M and 0.5 M, respectively (boundary conditions). Figure 5b is a representation of the solvent diffusivity versus the final salt concentration $D_{s}$(*c_salt_*) at a constant external pH (7).

To facilitate a direct comparison of solvent diffusivity, Figure 4b and Figure 5b have been designed with the same y scale.

Experimental results shown in Figure 4a and Figure 5a demonstrate that external pH and salt concentration have different impacts on the kinetics of the system reaching equilibrium.

As observed in Figure 4a, as the external pH changes, the hydrogel beads move toward the equilibrium point. It can be observed, from an experimental point of view, that the swelling kinetics of hydrogels is influenced by pH. From a macroscopic perspective, it can be observed that swelling kinetics is influenced by the external driving force. At a pH of 4.2, the system is close to the dissociation constant, resulting in slow swelling kinetics due to the limited dissociation of acidic groups. As the pH increases to 7.0, the driving force for dissociation strengthens, leading to faster-swelling kinetics. This effect becomes even more pronounced at higher pH levels, such as 11.0 and 13.0.

The influence of the driving force is also evident in Figure 5a, which illustrates the effect of salt concentration on the deswelling kinetics of hydrogel beads. The system, as a whole, consistently reaches equilibrium within approximately ten hours. However, as observed in the initial portion of the experimental data, the kinetics become macroscopically faster at higher salt concentrations. As discussed in Section 2.2, different salt concentrations exert a dual influence: they decrease the solvent’s chemical potential and induce the contraction of polymeric chains through electrostatic forces. Hydrogel beads immersed in solutions with varying ionic strengths are forced to desorb water and shrink due to the combined effects of these two phenomena. The observation that external stimuli influence swelling (or deswelling) kinetics is consistent with the literature data regarding swelling/deswelling kinetics at varying pH and salt concentrations, highlighting that greater variations in pH or salt concentration lead to faster macroscopic kinetics [32,33].

Figure 4a and Figure 5a show the experimental results together with the modeling results. Parameters obtained under steady-state conditions and summarized in Table 1 are used in the transient regime simulations without any further optimization. The new parameters used in the dynamic simulation are the diffusivities of the species present in the system. The ion diffusivities were taken from the literature (as reported in the modeling section), while the only parameter optimized in the simulation is the solvent diffusivity: $D_{s}=[\frac{m^{2}}{s}]$. The solvent diffusivity parameter was optimized individually for each simulation, and the resulting values are reported in Figure 4b and Figure 5b, respectively.

Globally, it can be observed that solvent diffusivity is linked to the expansion and contraction of the polymeric network (which is globally linked to the system deformation *J_D_*): a large polymeric mesh allows for easier solvent movements within the gels, while a more compact network hinders solvent diffusion.

In Figure 4b, at constant salt concentration, the value of solvent diffusivity varies with external pH. Specifically, it is lowest for a pH of 4.2, increases at a pH of 7 and 11, and decreases again at a pH of 13. This can be attributed, as discussed in Section 2.2 to the influence of external pH on the dissociation and association of functional groups. These processes lead to the expansion and contraction of the polymeric chain.

Starting from a pH of 7 at a salt concentration of 0.0015 M, beads were transferred to solutions at higher ionic strengths (0.01 M, 0.05 M and 0.1 M). As expected, the presence of salt induces polymeric chain contraction, leading to a decrease in solvent diffusivity with increasing ionic strength. However, at a salt concentration of 0.01 M, as reported in Figure 5b, the solvent diffusivity is higher than the one at the same pH but with a salt concentration of 0.0015 M, as reported in Figure 4b.

This behavior could be explained considering phenomena occurring at a scale smaller than the Debye length: (i) the shielding effect and (ii) electrostatic interactions.

At a pH of 7 and a salt concentration of 0.0015 M (initial conditions), all ionizable groups are fully dissociated, allowing for unrestricted solvent diffusion through the polymeric network. Increasing the ionic strength results in effective charge screening, leading to a rapid decrease in gel volume and a corresponding increase in solvent diffusivity.

At higher salt concentrations, competition for counter ions or entropic effects can mitigate the screening effect, resulting in a slower rate of gel contraction and a lower value of solvent diffusivity.

As observed in Figure 4a and Figure 5a, the model can depict the overall behavior of the gel with only one optimization parameter.

## 3. Conclusions

Polyelectrolyte hydrogels are a unique class of materials whose swelling behavior is influenced by the presence of functional groups on their polymeric chain. These pendant groups can dissociate and associate in solution, leading to the formation of “fixed groups” and “mobile ions”. The interplay of elastic, entropic and enthalpic forces governs the equilibrium behavior of these materials.

In this work, experimental and modeling approaches were used to investigate the steady state and the transient regime behavior of anionic hydrogels under varying pH and ionic strength conditions. The experimental protocol was designed to isolate the effects of the two external stimuli.

As regards the steady state, the results confirmed the previous findings of a bell-shaped pH-dependent swelling behavior, with increased swelling at intermediate pH values. However, this effect is mitigated by an increase in the ionic strength, resulting in a less pronounced pH dependence. This reduction in pH sensitivity is attributed to the combined effect of increased external solvent chemical potential and chain contraction due to electrostatic forces, which ultimately lead to hydrogel shrinkage.

The modeling results demonstrate the model’s ability to accurately describe the overall steady-state behavior of the hydrogel. With optimization of only three parameters for the initial salt concentration, the model becomes predictive in the other conditions.

The transient regime experiments demonstrate that both swelling and deswelling kinetics are influenced by external stimuli. Specifically, experimental data reveal that the swelling and deswelling kinetics become faster as the driving force increases, whether through changes in pH or salt concentration.

The model utilizes the same optimized parameters from the steady-state simulation with the addition of the diffusivity parameters. Ion’s diffusivities were taken from the literature, and the solvent diffusivity becomes the only optimization parameter for the transient regime simulation. The solvent diffusivity is optimized individually for each simulation, and the resulting optimized values are analyzed. Globally, the solvent diffusivity is linked to the expansion and contraction of the polymeric network, and its values are linked to the variation in pH and ionic strength. As expected, solvent diffusivity exhibits a bell-shaped behavior with varying external pH and decreases with increasing salt concentration. The highest value was observed at the lowest salt concentration, reflecting the electrostatic phenomena that occur under these conditions and vanish at higher ionic strength.

## 4. Materials and Methods

In this study, the experiments were performed using commercial beads (Orbeez^TM,^ Spin Master Ltd., 225 King Street West, Toronto, ON, Canada) made of sodium polyacrylate, a super absorbent polymer that can absorb 100 to 1000 times its mass in water. Sodium polyacrylate, with the chemical formula [-CH_2_-CH(COONa)-]_n_, is a long-chain copolymer consisting of alternate units of acrylic acid and sodium acrylate. Due to the presence of acrylic acid and its negatively charged carboxylic groups in the main chain, this copolymer is considered an anionic polyelectrolyte. The beads, in their dry configuration, have an initial mass (*M_Dry_*) of 10 mg and a radius of 1.15 mm. Hydrochloric acid (CAS 7647-01-0), sodium hydroxide (CAS 1310-73-2) and sodium chloride (CAS 7647-14-5) were purchased by Sigma Aldrich (Milan, Italy). Solutions with a pH lower and higher than 7 were prepared by adding a proper quantity of hydrochloric acid 0.1 M and sodium hydroxide 0.5 M and adjusting the pH until the desired value was reached. Solutions at different ionic strengths were prepared by adding a proper quantity of sodium chloride.

### 4.1. Swelling/Deswelling Experiments

The equilibrium behavior of the hydrogels was recorded by placing beads in solutions with different pH values (ranging from 2.0 to 14.0) and at three different salt concentrations (0.0015 M, 0.01 M and 0.5 M). The equilibrium mass of the beads in the current state (*M*) was recorded after approximately 200 h. The swelling ratio was calculated as the ratio between the mass in the current state (*M*) and the reference mass at the dry state (*M_Dry_*), where the mass in the current state (*M*) represents the total sum of the water and the polymer.

The ratio *M*/*M_Dry_* is then reported as $V/V_{Dry}=J_{D}$, considering the volume additivity: $V=V_{Water}+V_{Dry}=\rho_{water}\left(M-M_{Dry}\right)+V_{Dry}$. *J_D_* represents the system deformation, and the subscript “*D*” will be explained in the modeling section.

The dynamic behavior of the hydrogels was also recorded by monitoring their mass over time. Beads were first equilibrated in solution at pH = 2.0 and then transferred into the solution at a different pH; in particular, the dynamic behavior was recorded for a final pH equal to 4.2, 7.0, 11.0 and 13.0. The dynamic behavior for different ionic strengths was also recorded. In this case, beads were equilibrated into a solution at pH = 7.0 and concentration of salt = 0.0015 M and then transferred into solutions at the same pH but with different ionic strengths: 0.01 M, 0.05 M, and 0.5 M. In the presence of the new solution, gels began to swell (or to shrink, depending on the context) toward a new equilibrium point, and their mass (*M*) was recorded until the new equilibrium was reached.

### 4.2. Mechanical Compression Test

Mechanical compression tests were performed on the hydrogel’s beads at different hydration levels, i.e., at different polymer volume fractions *ϕ_2_* calculated as *V_Dry_*/*V*. The mechanical tests were performed using the Texture Analyzer (TA.XT Stable Micro System Ltd., Godalming, UK) equipped with a 5 kg_f_ (40 N) loading cell and using a cylindrical probe (diameter 20 mm). The probe vertically moves at 0.1 mm/s until a “trigger force” of 0.5 g_f_ was reached. The value of the “trigger force” was chosen not to deform the beads but to ensure contact between the top surface of the beads and the surface of the probe. Once the trigger force was reached, a deformation of 10% was applied moving the probe at 1 mm/s. The value of the deformation was chosen according to the Hertzian theory [13], which has been validated for deformation up to 1–10%. At the end of the test, once the value of the deformation imposed was reached, the probe returned to its initial position. During the experiment, values of the position of the probe and the force were recorded, the value of the wet radius (*R*) and of the applied force (*F*) were read. The deformation of the sphere (*δ*) was calculated as 0.1 *R*, and from Equation (2), the contact radius (2a), the reduced Young’s modulus (2b) (E*) and the Young’s modulus (2c) (*E*) were derived. The assumption of a perfectly incompressible isotropic material leads to the well-known relation *E* = 3*G*, so the shear modulus of the swollen hydrogel was calculated as reported in (2d). The equations used are reported in (2), according to the Hertzian relations [34]:

(2a) $$a=\sqrt{R\delta }$$

(2b) $$a={\left(\frac{3}{4}\times \frac{R}{E^{*}}F\right)}^{\frac{1}{3}}$$

(2c) $$\frac{1}{E^{*}}=\frac{1-\upsilon_{sphere}^{2}}{E_{sphere}}$$

(2d) $$G_{swollen}=E_{sphere}/3$$

where *a* is the contact radius, *R* is the particle radius, *δ* is the deformation of the sphere and flat plane, *F* is the applied force in the normal direction, E* is the reduced Young’s modulus of the contact, *E* is the Young’s modulus, and *v* is the Poisson ratio.

## 5. Modeling

As discussed in the introduction, the following model couples mass transport with solid mechanics. The two phenomena have a different reference configuration: mass transport is usually referred to spatial coordinates, following a Eulerian Approach; conversely, solid mechanics is usually referred to material coordinates according to a Lagrangian approach, hence the need to make the coordinate system uniform and use a proper and single reference frame. Generally, dealing with solid mechanics in spatial coordinates is problematic; for this reason, all the following equations will refer to the reference coordinates. As explained by Caccavo and Lamberti, 2017 [8] and De Piano et al., 2023 [9], the choice of the frame of reference should be arbitrary; in this case, the use of the rubber elasticity theory in the elastic contribution of the Helmholtz Free energy makes it necessary to use the hydrogel in its dry configuration as a frame of reference. However, while the steady-state behavior could be described on the dry configuration, for the transient behavior, a change from the dry to the “swelling free state” reference should be performed to avoid numerical singularities at the initial point. In the present work, since both the steady and the unsteady state behavior will be studied, two different reference configurations will be used. In particular, the material coordinates on the dry state will be identified with the capital letter with the subscript “*D*” (e.g., for the concentration $C_{D}\;\left[mol/m_{Dry}^{3}\right]$); the material coordinates on the swelling free state will be identified with the capital letter without any subscript (e.g., for the concentration $C\;\left[mol/m_{SFS}^{3}\right]$); the spatial coordinates will be identified with the lowercase letter without any subscript (e.g., for the concentration $c\;\left[mol/m^{3}\right]$). Readers could refer to Caccavo and Lamberti, 2017 [22,35], Caccavo et al., 2018 and De Piano et al., 2023 [24] for further information.

### 5.1. Phenomenology

The aim of this study is to describe the behavior of an anionic hydrogel in solution when exposed to two external stimuli: changes in pH and ionic strength. This paragraph will provide a general description of the system and the relationships between the involved species, along with the principal hypotheses. The hydrogel beads are immersed in a solvent (“*s*”) containing various dissolved species, including protons (“*H*^+^”), hydroxide ions (“*OH*^−^”), sodium cations (“+”) and chlorine anions (“−”). These species are referred to as “mobile” species. Meanwhile, the polymeric network comprises several functional groups (“AH”) characteristic of the anionic gel, which dissociate (3a) at a specific pH (approximately equal to 4), resulting in charged groups (“*A*^−^”) along the chain. At pH levels above 7, these groups can form ion pairs with sodium ions (“ANa”) (3b).

(3a) $$AH⇄A^{-}+H^{+}$$

(3b) $$ANa⇄A^{-}+{Na}^{+}$$

These groups represent the “fixed” species and are related by the global balance reported in Equation (6e). In this expression, $f/\Omega_{m}$ is defined as the concentration of total functional groups present on the chain in the dry state $\left[mol/m_{Dry}^{3}\right]$.

The sodium ions involved in the association reaction (3a) are those provided by the sodium hydroxide at pH levels above 7.

Hydrogel beads immersed in a solvent are subjected to free swelling, which is mathematically described by a homogeneous deformation gradient, along with the assumption of isotropic stretches.

### 5.2. Free Energy Imbalance and Constitutive Equations

Similar to the approach taken in references [8,9], starting from non-equilibrium thermodynamics [36] and using the appropriate expression for the Helmholtz Free Energy, it is possible to derive the constitutive equations that relate stress to the deformation and chemical potentials to the concentrations.

(4) $$\frac{dA_{R}}{dt}-\overset{̿}{P}∶\dot{\overset{̿}{F}}-\sum_{i}\left(\mu_{i}\frac{dC_{i}}{dt}\right)+\sum_{i}\left(\bar{h_{i}}\cdot \bar{\nabla }\mu_{i}\right)\leq 0$$

In Equation (4), the term *A* represents the relaxed version of the Helmholtz free energy density, $\overset{̿}{P}$ is the Piola stress tensor, $\overset{̿}{F}$ is the deformation gradient, $\mu_{i}$ is the chemical potential, *C_i_* is the molar concentration and *h_i_* is the flux of the ith species.

For polyelectrolytes, the Helmholtz Free Energy is the sum of four contributions. The mechanical contribution to the free energy is limited to an elastic contribution, *A^El^*, which is expressed using the affine network theory [37]. The interaction between the polymer and solvent, *A^Mix^*, is described through Flory Huggins mixing theory [38]. Additionally, the presence of “mobile” ions and “fixed” groups, as described earlier, contributes to the ionization term *A^Ion^* and to the dissociation term *A^Diss^*, respectively [25,39,40]. The relaxed version of the Helmholtz free energy also accounts for the volumetric constraint. Further detail can be found in references [16,24].

### 5.3. Steady State’s Modeling

Regarding the mechanics, the linear momentum balance is expressed using a *quasistatic* approach, and the Piola stress tensor ($\overset{̿}{P}$) should be derived from the derivative of the Helmholtz free energy with respect to the deformation.

From the affine network theory and isotropic deformation: $\overset{̿}{P}=\overset{̿}{I}\left(G\;\left(\lambda -\frac{1}{\lambda }\right)-p\lambda^{2}\right)$, where $\overset{̿}{I}$ is the identity matrix, *G* is the dry shear modulus of the polymer, *λ* is the stretch with respect to the dry state, and *p* is the pressure. The steady state describes a “stress free state” condition, where $\overset{̿}{P}=\overset{̿}{0}$; therefore, Equation (6a) must hold.

In Equation (6b), the volumetric constraint is reported, relating the system deformation (*J_D_*) to the solvent concentration *C_sD_* and vice versa, where $\Omega_{S}$ is the solvent molar volume.

Under equilibrium conditions, reactions (3a) and (3b) are characterized by the relationship presented in Equation (6f,g), where *K_a_* and *K_Na_* are the equilibrium constants. *K_a_* is the acidic dissociation constant; *K_Na_* represents the dissociation constant of the ion pairs “ANa”.

At the steady state conditions, another equation must be satisfied, specifically the equation presented in Equation (6d), which describes a condition of global electro-neutrality.

The thermodynamic criterion of the phase equilibrium $\mu_{i}^{\alpha }=\mu_{i}^{\beta }$ allows to establish a relation between the internal and external chemical potential for all species. The expressions of the external chemical potentials are provided below (5). The external chemical potential of the solvent (5a) is written under the assumption of a dilute system.

(5a) $${\left(\mu_{s}\right)}^{ext}=-RT\Omega_{s}\left(c_{H^{+}}^{ext}+c_{+}^{ext}+c_{-}^{ext}+c_{{OH}^{-}}^{ext}\right)$$

(5b) $${\left(\mu_{i}\right)}^{ext}=RT\ln\left(\frac{c_{i}^{ext}}{c_{i}^{rif}}\right)$$

The mathematical system that encompassed the equality of the chemical potentials can be solved as described in our previous works [16,24].

This study adopts a simpler approach, following Marcombe et al. (2010) [25], to derive a relationship between concentrations rather than chemical potentials. In particular, by applying the electro-neutrality condition within the framework of free energy imbalance, the Donnan Equation (6h–j) can be derived, establishing a relationship between concentrations of “mobile” species. Details are reported in Appendix A.

The equations solved under steady-state conditions in the reference configuration of the dry state are presented in the following Equation (6a–j), along with the variables for which the solutions are obtained: *p*, *λ*, *C_sD_*, $C_{A^{-}D}$, *C_AHD_*, *C_ANaD_*, $C_{H^{+}D}$, *C_+D_*, $C_{{OH}^{-}D}$.

(6a) $$G\;\left(\lambda -\frac{1}{\lambda }\right)-p\lambda^{2}=0$$

(6b) $$J_{D}-\left(1+\Omega_{S}C_{sD}\right)=0$$

(6c) $${\left(\mu_{s}\right)}_{int}-{\left(\mu_{s}\right)}_{ext}=0$$

(6d) $$C_{A^{-}D}=C_{H^{+}D}+C_{+D}-C_{{OH}^{-}D}-C_{-D}$$

(6e) $${\left(f/\Omega_{m}\right)}_{D}=C_{AHD}+C_{ANaD}+C_{A^{-}D}$$

(6f) $$k_{Na}=\frac{C_{A^{-}D}C_{+D}}{C_{ANaD}}\frac{1}{\left(1+\Omega_{s}C_{sD}\right)}$$

(6g) $$k_{a}=\frac{C_{H^{+}D}C_{A^{-}D}}{C_{AHD}}\frac{1}{\left(1+\Omega_{s}C_{sD}\right)}$$

(6h) $$\frac{C_{+D}}{c_{+}^{ext}}=\frac{C_{H^{+}D}}{c_{H^{+}}^{ext}}$$

(6i) $$\frac{C_{{OH}^{-}D}}{c_{{OH}^{-}}^{ext}}=\frac{c_{H^{+}}^{ext}}{C_{H^{+}D}}{\left(1+\Omega_{s}C_{sD}\right)}^{2}$$

(6j) $$\frac{C_{-D}}{c_{-}^{ext}}=\frac{c_{H^{+}}^{ext}}{C_{H^{+}D}}{\left(1+\Omega_{s}C_{sD}\right)}^{2}$$

The model is described by six parameters: the elastic modulus *G_Dry_*, the association and dissociation constant *K_a_* and *K_Na_*, the number of total functional groups *f* and the Flory Huggins interaction parameter *χ_12_*.

#### Implementation at Steady State

The implementation of the seven algebraic Equation (6a–j) has been carried out in MATLAB (R2022a). From Equation (6d–j), an explicit expression for the concentration of protons $C_{H^{+}D}$, a function of the solvent concentration is derived. This expression provides a fourth-order equation that can be substituted into the expression of the chemical potential of the solvent, as shown in Equation (6c). The system formed by Equation (6a–c) is numerically solved using the function *fsolve*, which is a nonlinear system solver.

In the MATLAB implementation, the elastic modulus is obtained from the independent mechanical experiments, while the dissociation constant is taken from reference [15]. For the association constant, a medium value close to the acidic dissociation value of carboxylic acid was selected (further details will be provided in the results and discussion section).

The Flory–Huggins parameter is defined as a function of the internal pH ${(pH}_{int})$, which is calculated based on the internal protons concentration, resulting in the definition of two parameters: *α* and *β* Equation (1).

The optimization of three parameters (*f*, *α* and *β*) was performed using mathematical optimization. In particular, a Sum of Squared Errors (SSE) function was generated by summing the squared differences between the experimental data obtained at a salt concentration of 0.0015 M (refer to the materials and methods section) and the predicted value of the swelling ratio. The SEE was minimized by varying the three parameters using the *fmincon* and *patternsearch* methods.

### 5.4. Transient Regime’s Modeling

The transient model, as expressed before, cannot be solved on a Lagrangian frame of reference that corresponds to the dry state since the Flory–Huggins equation shows a singularity at that point. The problem is normally avoided by considering an isotropic volumetric deformation (*J*_0_) from the dry to a “swelling free state” (SFS), which became the Lagrangian frame of reference [22,24,35].

In terms of mechanics, the Piola stress tensor, similar to its derivation in the steady state, is obtained from the derivative of the Helmholtz free energy with respect to the deformation, while accounting for swelling free state coordinates. This expression for the Piola stress tensor is then substituted in Equation (8a), which uses a quasistatic approximation, and solved. Additionally, the change in coordinates (from dry to SFS) incorporates the volumetric constraint, as expressed in Equation (8b). For unsteady-state modeling, the reactions described in Equation (3a,b) are written considering the reaction rate, with *K_a_* defined as *k*_1_/*k*_2_ and *K_Na_* = *k*_4_/*k*_3_, as indicated in Equation (7a,b).

(7a) $${\left(-r_{a}\right)}_{1}=k_{1}c_{AH}-k_{2}c_{A^{-}}c_{H^{+}}$$

(7b) $${\left(-r_{a}\right)}_{2}=k_{3}c_{+}c_{A^{-}}-k_{4}c_{ANa}$$

Mass balances are presented in Equation (8c–j). The solvent, hydroxide ions and chlorine ions exhibit diffusive transport, while protons and sodium ions are also involved in reactions. The assumption of electro-neutrality is only valid at a steady state; therefore, in the unsteady-state model, the concentrations of “fixed” species are expressed considering the reaction rate in which they participate.

The diffusive flux for the solvent is expressed as the gradient of the chemical potential (8c) following the approach of Caccavo et al. [22]. For Equation (8g–j), the diffusive flux is described as the gradient of the concentration according to Fick’s law.

(8a) $$\bar{\nabla }\cdot \overset{̿}{P}=\bar{0}$$

(8b) $$J=\frac{{1+\Omega }_{s}C_{s}J_{0}}{J_{0}}$$

(8c) $$\frac{\partial C_{s}}{\partial t}=-\bar{\nabla }\cdot \left(-D_{s}\overset{̿}{I}\cdot \bar{\nabla }\mu_{s}\right)$$

(8d) $$\frac{\partial C_{A-}}{\partial t}=+{\left(-r_{a}\right)}_{1}$$

(8e) $$\frac{\partial C_{AH}}{\partial t}=-\left({{\left(-r_{a}\right)}_{2}-\left(-r_{a}\right)}_{1}\right)$$

(8f) $$\frac{\partial C_{ANa}}{\partial t}=-{\left(-r_{a}\right)}_{2}$$

(8g) $$\frac{\partial C_{H^{+}}}{\partial t}=-\bar{\nabla }\cdot \left(-D_{H^{+}}\overset{̿}{I}\cdot \bar{\nabla }C_{H^{+}}\right)+{\left(-r_{a}\right)}_{1}$$

(8h) $$\frac{\partial C_{+}}{\partial t}=-\bar{\nabla }\cdot \left(-D_{+}\overset{̿}{I}\cdot \bar{\nabla }C_{+}\right)+{\left(-r_{a}\right)}_{2}$$

(8i) $$\frac{\partial C_{{OH}^{-}}}{\partial t}=-\bar{\nabla }\cdot \left(-D_{OH^{-}}\overset{̿}{I}\cdot \bar{\nabla }C_{OH^{-}}\right)$$

(8j) $$\frac{\partial C_{-}}{\partial t}=-\bar{\nabla }\cdot \left(-D_{-}\overset{̿}{I}\cdot \bar{\nabla }C_{-}\right)$$

The term $D_{i}\overset{̿}{I}$ represents the mobility tensor $\;\overset{̿}{D}$, as explained in reference [22].

#### Implementation at Transient Regime

The implementation of the ten partial differential Equation (8a–j) was carried out on COMSOL Multiphysics 6.1.

The implementation was performed in a 1D system in spherical coordinates:

The ten PDE Equation (8a–j) were written using their weak formulation, as shown in Equation (9a–j):

(9a) $$0=-\int_{0}^{\bar{R}}4\pi R^{2}\left(\overset{̿}{P}\cdot \left(\;\bar{\nabla }\tilde{u}\right)\right)dr$$

(9b) $$0=-\int_{0}^{\bar{R}}4\pi R^{2}\left(J-\frac{{1+\Omega }_{s}C_{s}J_{0}}{J_{0}}\right)\tilde{p}\;dr$$

(9c) $$0=-\int_{0}^{\bar{R}}4\pi R^{2}\left(\frac{\partial C_{s}}{\partial t}\right)\tilde{c}dr+\int_{0}^{\bar{R}}4\pi R^{2}\left({\bar{h}}_{s}\cdot \left(\;\bar{\nabla }\tilde{c}\right)\right)dr$$

(9d) $$0=-\int_{0}^{\bar{R}}4\pi R^{2}\left(\frac{\partial C_{A^{-}}\;}{\partial t}\right)\tilde{c}dr+\int_{0}^{\bar{R}}4\pi R^{2}{\left(-r_{a}\right)}_{1}\tilde{c}dr$$

(9e) $$0=-\int_{0}^{\bar{R}}4\pi R^{2}\left(\frac{\partial C_{AH}\;}{\partial t}\right)\tilde{c}dr-\int_{0}^{\bar{R}}4\pi R^{2}({\left(-r_{a}\right)}_{2}-{\left(-r_{a}\right)}_{1})\tilde{c}dr$$

(9f) $$0=-\int_{0}^{\bar{R}}4\pi R^{2}\left(\frac{\partial C_{ANa}\;}{\partial t}\right)\tilde{c}dr+\int_{0}^{\bar{R}}4\pi R^{2}{\left(-r_{a}\right)}_{2}\tilde{c}dr$$

(9g) $$0=-\int_{0}^{\bar{R}}4\pi R^{2}\left(\frac{\partial C_{H^{+}}}{\partial t}\right)\tilde{c}dr+\int_{0}^{\bar{R}}4\pi R^{2}\left({\bar{h}}_{H^{+}}\cdot \left(\;\bar{\nabla }\tilde{c}\right)\right)dr+\int_{0}^{\bar{R}}4\pi R^{2}{\left(-r_{a}\right)}_{1}\tilde{c}dr$$

(9h) $$0=-\int_{0}^{\bar{R}}4\pi R^{2}\left(\frac{\partial C_{+}}{\partial t}\right)\tilde{c}dr+\int_{0}^{\bar{R}}4\pi R^{2}\left({\bar{h}}_{+}\cdot \left(\;\bar{\nabla }\tilde{c}\right)\right)dr+\int_{0}^{\bar{R}}4\pi R^{2}{\left(-r_{a}\right)}_{2}\tilde{c}dr$$

(9i) $$0=-\int_{0}^{\bar{R}}4\pi R^{2}\left(\frac{\partial C_{{OH}^{-}}}{\partial t}\right)\tilde{c}dr+\int_{0}^{\bar{R}}4\pi R^{2}\left({\bar{h}}_{{OH}^{-}}\cdot \left(\;\bar{\nabla }\tilde{c}\right)\right)dr$$

(9j) $$0=-\int_{0}^{\bar{R}}4\pi R^{2}\left(\frac{\partial C_{-}}{\partial t}\right)\tilde{c}dr+\int_{0}^{\bar{R}}4\pi R^{2}\left({\bar{h}}_{-}\cdot \left(\;\bar{\nabla }\tilde{c}\right)\right)dr$$

where $\tilde{c_{i}},\tilde{u},\tilde{p}$ represent the test functions (Lagrange quadratic shape function) of the concentration of the ith species, the deformation and the pressure.

The unsteady state simulations were conducted by varying two external stimuli: starting at a pH of 2.0 (SFS for pH varying simulations) with a salt concentration of 0.0015 M and changing the final pH (4.2, 7.0, 11.0 and 13.0), and beginning at a pH of 7.0 (SFS for ionic strength varying simulations) and a salt concentration of 0.0015 M and varying the ionic strength (0.01 M, 0.05 M and 0.5 M).

The values of the variables at these initial conditions were derived from steady-state simulations at an initial salt concentration of 0.0015 M and initial pH values of 2.0 and 7.0, respectively.

Since the initial state is the SFS, the hydrogel is not yet deformed: $\bar{u}=0$.

The boundary conditions are established at the interface with the external bath at the radius R. On this boundary, equilibrium is assumed between the internal and the external phase, allowing for the application of Equation (6c–j) with the appropriate reference adjustments. Here, it can freely deform.

The ion diffusivities were taken from reference [41], in particular: $D_{H^{+}}=9.310\times 10^{-9}[\frac{m^{2}}{s}]$, $D_{+}=1.330\times 10^{-9}[\frac{m^{2}}{s}]$, $D_{OH^{-}}=5.270\times 10^{-9}[\frac{m^{2}}{s}]$, $D_{-}=2.030\times 10^{-9}[\frac{m^{2}}{s}]$.

The solvent diffusivity, $D_{s}$, was used as an optimization parameter.

## Figures and Tables

**Table 1:** Parameters optimized for the steady-state simulation.

Parameters		Source
*K_Na_*	10^−1^	Literature ([31])
*G_Dry_*	140 kPa	Independent experiment (Section Mechanical Compression Test)
*K_a_*	10^−3.8^	Optimization ^1^
*f*	0.14	Optimization
*χ*_12_	*α* = 0.38	Optimization
	*β* = 0.78	Optimization

^1^ Close to pKa = 4, acid dissociation constant.

## Data Availability

The raw data supporting the conclusions of this article will be made available by the authors on request.

## Appendix A. Derivation of the Donnan Equations

As explained in the modeling section (Section 5.3), the system could be solved using the equality of chemical potentials, as performed in previous work. However, this work employs a different approach, deriving the Donnan equations from the free energy Imbalance. The key steps are summarized below. Starting from the relaxed form of the Helmholtz free energy, its time derivative with respect to all relevant variables can be derived using Equation (A1):

(A1) $$\begin{array}{cc} \frac{{dA}_{R}}{dt}=\frac{{dA}_{R}}{d\overset{̿}{F}}:\frac{d\overset{̿}{F}}{dt} & +\frac{{dA}_{R}}{dC_{s}}\frac{dC_{s}}{dt}+\frac{{dA}_{R}}{dC_{H^{+}}}\frac{dC_{H^{+}}}{dt}+\frac{{dA}_{R}}{dC_{+}}\frac{dC_{+}}{dt}\\ & +\frac{{dA}_{R}}{dC_{-}}\frac{dC_{-}}{dt}+\frac{{dA}_{R}}{dC_{{OH}^{-}}}\frac{dC_{{OH}^{-}}}{dt}+\frac{{dA}_{R}}{dp}\frac{dp}{dt} \end{array}$$

By substituting Equation (A1) into the free energy imbalance Equation (3) and rearranging, Equation (A2) could be obtained:

(A2) $$\begin{array}{cc} \left(\frac{{dA}_{R}}{d\overset{̿}{F}}-\overset{̿}{P}\right):\frac{d\overset{̿}{F}}{dt} & +\left(\frac{{dA}_{R}}{dC_{s}}-\mu_{s}\right)\frac{dC_{s}}{dt}+\left(\frac{{dA}_{R}}{dC_{H^{+}}}-\mu_{H^{+}}\right)\frac{dC_{H^{+}}}{dt}+\left(\frac{{dA}_{R}}{dC_{+}}-\mu_{+}\right)\frac{dC_{+}}{dt}\\ & +\left(\frac{{dA}_{R}}{dC_{-}}-\mu_{-}\right)\frac{dC_{-}}{dt}+\left(\frac{{dA}_{R}}{dC_{{OH}^{-}}}-\mu_{{OH}^{-}}\right)\frac{dC_{{OH}^{-}}}{dt}+\frac{{dA}_{R}}{dp}\frac{dp}{dt}\\ & +\sum_{i}\left(\bar{h_{i}}\cdot \bar{\nabla }\mu_{i}\right)\leq 0 \end{array}$$

Deviating from our previous work and following the approach of Marcombe [25], the electroneutrality condition (A3) was used. This equation is then substituted into (A2):

(A3) $$\frac{dC_{H^{+}}}{dt}=\frac{dC_{A^{-}}}{dt}+\frac{dC_{-}}{dt}+\frac{dC_{{OH}^{-}}}{dt}-\frac{dC_{+}}{dt}$$

By rearranging the resulting equation, and in order to satisfy the inequality condition, Equation (A4a–c) are derived:

(A4a) $$\frac{{dA}_{R}}{dC_{H^{+}}}+\frac{{dA}_{R}}{dC_{{OH}^{-}}}=\mu_{H^{+}}+\mu_{{OH}^{-}}$$

(A4b) $$\frac{{dA}_{R}}{dC_{+}}-\frac{{dA}_{R}}{dC_{H^{+}}}=\mu_{+}-\mu_{H^{+}}$$

(A4c) $$\frac{{dA}_{R}}{dC_{H^{+}}}+\frac{{dA}_{R}}{dC_{-}}=\mu_{H^{+}}-\mu_{-}$$

By considering the equilibrium conditions and imposing the equality of chemical potentials between internal and external phases (A5), the Donnan Equation (6h–(6j) can be derived:

(A5a) $$\mu_{H^{+}}+\mu_{{OH}^{-}}={\left(\mu_{H^{+}}\right)}^{ext}+{\left(\mu_{{OH}^{-}}\right)}^{ext}$$

(A5b) $$\mu_{+}-\mu_{H^{+}}={\left(\mu_{+}\right)}^{ext}-{\left(\mu_{H^{+}}\right)}^{ext}$$

(A5c) $$\mu_{H^{+}}-\mu_{-}={\left(\mu_{H^{+}}\right)}^{ext}+{\left(\mu_{-}\right)}^{ext}$$
